# Energy-Efficient IoT-Based Light Control System in Smart Indoor Agriculture

**DOI:** 10.3390/s23187670

**Published:** 2023-09-05

**Authors:** Oussama Hadj Abdelkader, Hadjer Bouzebiba, Danilo Pena, António Pedro Aguiar

**Affiliations:** Research Center for Systems and Technologies (SYSTEC), ARISE, Faculdade de Engenharia, Universidade do Porto, Rua Dr. Roberto Frias, s/n, 4200-465 Porto, Portugal; bouzebiba.hadjer@gmail.com (H.B.); danilopena.ba@gmail.com (D.P.); pedro.aguiar@fe.up.pt (A.P.A.)

**Keywords:** controlled environment agriculture, distributed control, internet of things, remote control, smart indoor farming, wireless sensor network

## Abstract

Indoor agriculture is emerging as a promising approach for increasing the efficiency and sustainability of agri-food production processes. It is currently evolving from a small-scale horticultural practice to a large-scale industry as a response to the increasing demand. This led to the appearance of plant factories where agri-food production is automated and continuous and the plant environment is fully controlled. While plant factories improve the productivity and sustainability of the process, they suffer from high energy consumption and the difficulty of providing the ideal environment for plants. As a small step to address these limitations, in this article we propose to use internet of things (IoT) technologies and automatic control algorithms to construct an energy-efficient remote control architecture for grow lights monitoring in indoor farming. The proposed architecture consists of using a master–slave device configuration in which the slave devices are used to control the local light conditions in growth chambers while the master device is used to monitor the plant factory through wireless communication with the slave devices. The devices all together make a 6LoWPAN network in which the RPL protocol is used to manage data transfer. This allows for the precise and centralized control of the growth conditions and the real-time monitoring of plants. The proposed control architecture can be associated with a decision support system to improve yields and quality at low costs. The developed method is evaluated in emulation software (Contiki-NG v4.7),its scalability to the case of large-scale production facilities is tested, and the obtained results are presented and discussed. The proposed approach is promising in dealing with control, cost, and scalability issues and can contribute to making smart indoor agriculture more effective and sustainable.

## 1. Introduction

Technological adoption in agriculture is slow in comparison to other industrial sectors and varies widely depending on the type of the cultivation method and the region. In fact, many agricultural tasks still rely on manual labor and traditional low-tech methods, resulting in low productivity. In general, the current situation is still far from satisfying the goals of Agriculture 4.0 [1], which aims to transform agriculture into an efficient, data-driven industry that uses the latest technologies to optimize production and promote sustainability [2].

That being said, there are several recent research advancements in the agricultural sector that provide a positive outlook aligning with the vision of Agriculture 4.0. For instance, there is the increasing use of sensing technologies and data analytics to make informed decisions in agriculture. Furthermore, numerous companies are developing groundbreaking products and services that exploit recent technologies; when these improve in terms of accessibility and affordability, their adoption in the agricultural sector will increase [2]. These technologies comprise internet of things (IoT) [3,4,5,6], robotics [7,8], artificial intelligence (AI) [9,10], blockchain [11], remote sensing technologies [12,13,14], image processing [15,16], and vertical farming [17,18]. The objectives are to automate the production process and build decision support systems [19] that use sensors to collect data on environmental conditions, soil condition, and plant growth, in addition to devices that control irrigation, fertilization, lighting, and other tasks. IoT plays a paramount role in smart agriculture as it ensures connectivity and data exchange between the different devices that collect, send, process, and use data in the monitoring and control of various agricultural processes [20,21]. The benefits of this technology appear in large-scale farms where huge numbers of data on different agricultural products need to be collected and processed in a central server according to the user’s requirements. AI can be used in decision support systems [19] to analyze the collected data, find the relation between the variables of interest [22], and make predictions [15,23]. It can also be used in robots to automate tasks such as planting, irrigation, and harvesting [10,24], which reduces labor, waste, and time consumption and improves efficiency and precision. Blockchain can be used in agriculture to ensure that the food is produced ethically, improves transparency, and reduces fraud [11]. New sensing technologies and image processing methods improve accuracy in data acquisition and reduce the need for destructive analyses [25,26]. Vertical farming consists of growing plants vertically on shelves in controlled indoor environments with artificial lighting. It increases land use efficiency, supports circular economy, and reduces environmental impacts [17]. In this way, farmers and stakeholders can make informed and optimized decisions, adapt tasks to specific products, increase productivity and profitability, conserve resources, and plan future productions. This revolution in agriculture is an exciting opportunity to address the most pressing challenges of food security, resource depletion, and climate change that are currently facing the world [2].

Motivated by the above considerations, the case studied in this article is the distributed remote lighting control in large-scale vertical farms or plant factories where artificial lights are used to provide the optimal light intensities needed by plants throughout their life. Light-emitting diodes (LEDs) are proven to be the best artificial light source used in indoor agriculture [27]. In this study, it is supposed that three types of LEDs are used to provide different portions of the light spectrum, the light in the PAR (photosynthetically active radiation) region, the far-red light, and the UV (ultraviolet) light. These lights are controlled by a set of microcontrollers, each one of them is used to monitor the growth conditions in a specific growth chamber. Pulse width modulation (PWM) method is implemented in each microcontroller to dim the LEDs and change the obtained light intensities to the required levels. An additional microcontroller is used as a server to supervise all the growth chambers together from one place. A distributed control architecture is implemented in the server to remotely control the growth lights in multiple growth chambers by communicating with the microcontrollers in each one of them. The control architecture should have a low computational cost to avoid increasing the latency of the network and facilitate the data handling. The set of microcontrollers used in this case constructs a 6LoWPAN (IPv6 over low-power wireless personal area networks) network, which means that it exchanges data packets using the IPv6 protocol. The communication between the microcontrollers in this network can be subject to multiple constraints on data transmission, processing time, and energy consumption. RPL (routing protocol for low-power and lossy networks) protocol is used to transfer data in this kind of constrained network thanks to its low power consumption and its specific design for multi-hop and multi-point communications. In brief, the main contributions of the paper are the proposed architecture that improves the energy-efficiency of the plant factory by using economic LED lights, an energy-efficient PWM method instead of amplitude modulation for light dimming, a low computational cost-control method, and a 6LoWPAN network with the RPL routing protocol for constrained networks to perform tasks remotely.

The remainder of this article is organized as follows: Section 2 presents related works and similar IoT and control systems developed previously in the agricultural field. Section 3 explains the plant light requirements and shows why the automation of light control is needed. Section 4 shows the proposed control architecture and provides details about its different components and the used techniques. Section 5 discusses the implementation of the system and evaluates the obtained results. Finally, Section 6 concludes the article and provides hints about future research.

## 2. Literature Review

Smart indoor agriculture has recently attracted significant attention thanks to its ability to produce high-quality crops using fewer resources [17]. Artificial grow lights represent the key component of indoor agriculture; they play the critical role of providing light energy needed by plants for their growth and development processes [28,29]. Nevertheless, controlling the grow lights manually can be imprecise, time-consuming, and laborious, especially in large production facilities containing multiple products in different developmental stages. In this case, the automation of light control and its remote operation from a server become a necessity [30]. Furthermore, the advantages of automation and remote control appear in plant factories where fine crops are produced all year round in controlled cultivation environments [31].

As previously mentioned, using the latest technologies to make the agricultural field more efficient and “smarter” appears to be a very promising solution to the challenges of resource depletion and increasing demand [17,30]. Moreover, converting usual agricultural production to an indoor process where the growth conditions are controlled is expected to reduce the environmental impacts arising from farming practices and protect the crop from diseases, pests, and bad weather [18,28]. It will also enable us to exploit non-arable lands in the areas covered by snow, rocks, or sand in indoor agricultural production to cover the food demand in these areas and reduce importation costs. However, indoor farming facilities suffer from the high energy consumption problem, and this energy is consumed in the management of the indoor growth conditions [32,33]. State of the art contributions in indoor farming are mostly focused on finding solutions to this energy consumption problem [33,34,35]. In this article, we propose to continue in the same line by presenting an energy-efficient method based on IoT to remotely control the grow lights in indoor production facilities.

Several studies have explored the use of IoT technologies in smart agriculture, and general reviews about the different contributions, challenges, and research directions in this field have been proposed by [5,6,20,21,36,37]. Furthermore, many applications of IoT in the agricultural field can be found in the literature ranging from controlling tasks like irrigation [38], fertilization [39], harvesting [10], and other operations [8,24] to plant growth monitoring tasks [40,41], farm management systems [42,43], indoor farming systems [44,45,46,47], and the management of the food production chain [48]. The widespread use of IoT in agriculture is a result of its ability in data handling, its easy installation, and its decreasing cost. Moreover, many adaptations of existing IoT technologies to agricultural applications have been proposed to improve, for instance, data handling [49,50,51], the routing protocol [52,53,54], and energy efficiency [49,55].

The automatic grow light control problem was investigated by many researchers. As a result, different solutions were proposed such as a digital control system based on the PI (proportional integral) controller of the PWM signal presented in [56], a grow light control method for aeroponic systems based on random forest classification and sensor fusion described in [57], a control method combining grow lights and natural light that turns the lights on and off whenever natural light is not sufficient [58,59], a hybrid control method to control the PWM signal of LED grow lights [60], and a pulse lighting control method presented in [61] to find the best light exposure durations for plants in indoor farming. In brief, various methods to automatically control the grow lights were proposed for the goal of effectively managing lighting conditions and improving the yields and product quality. However, more research is needed to determine the optimal control strategies and technologies for different crop types and growth conditions, especially in large scale plant factories.

## 3. Plants Light Requirements

Light is the source of energy that plants use for their growth and development processes. It is therefore considered a very critical factor when growing plants in indoor environments. Thus, understanding plants’ light requirements is necessary for successful cultivation. Plants need very specific light conditions for optimal growth; these conditions include light intensity, light quality, light duration, and light source. Understanding and optimizing these factors can help to ensure a healthy and high-yielding plant growth, which results in a good final product. The light intensity received by plants is measured in photon flux density (PFD) (μmol/m${}^{2}$s) in the photosynthetically active radiation (PAR) region, which represents the spectrum region used for photosynthesis. The light intensity required by plants changes depending on the plant species, their growth stage, and some environmental factors like temperature and humidity. In general, the PAR light intensity required for growth is at least 100–200 μmol/m${}^{2}$s [62]. Light quality refers to the wavelengths of light spectrum that plants receive. It is known that red and blue wavelengths are used for photosynthesis with different amounts depending on the species and the growth stage. More specifically, blue light is needed more in the vegetative growth stage, while red light is needed more for flowering and fruiting. Light duration, also known by photoperiod, refers to the duration of plants’ light exposure per day. In general, the photoperiod is about 12–16 h per day, and it also varies depending on the species and the growth stage. By adjusting the photoperiod, developmental processes like germination or flowering can be triggered. Natural sunlight contains all the mentioned requirements, and it is therefore the ideal light source for plants. However, in the case of indoor farming, LED lights are gaining in popularity thanks to their energy-efficiency, durability, and ability to provide the necessary wavelengths of light.

### 3.1. Photosynthetically Active Radiation

PAR is the portion of the light spectrum used by plants in the photosynthesis process. PAR is also the visible light spectrum which contains wavelengths between approximately 400 nm and 700 nm. PAR is measured in PFD, which means the number of photons emitted on a unit area per unit of time. Plants convert PAR light energy into chemical energy in the form of glucose and other sugars through the photosynthesis process. In general, a minimum PAR intensity of around 100–200 μmol/m${}^{2}$s is required for vegetative growth, and 400–600 μmol/m${}^{2}$s or more is required for flowering and fruiting [62]. The optimal PAR intensities for a particular plant species and growth stage can be determined through experimentation and monitoring of plant growth and development under different intensity values.

### 3.2. Far Red Light Effect

Far-red is at the upper edge of the visible spectrum with a wavelength range of 700–750 nm. This kind of light can penetrate through dense canopies to reach the smaller plants. In this way, far-red light can stimulate stem elongation in these plants and push them to grow towards the light direction to get the other wavelengths, it is a phenomenon that can be easily observed in nature [63]. far-red light, with small exposure duration and intensities in the range of 5–20 μmol/m${}^{2}$s, can be beneficial for some plants that are produced for their stems. However, excessive exposure to far-red light can also cause a decrease in leaf size, chlorophyll content, and nutritional value of plants. Therefore, the exposure duration and the intensity of the far-red light should be precisely controlled to match the optimal intensity required by the produced species depending on its growth stage.

### 3.3. Ultraviolet (UV) Light Effect

UV light is at the range of 100–400 nm in the non-visible spectrum of light. UV light can stimulate the production of important chemical compounds like flavonoids and alkaloids in plants [27]. Flavonoids are responsible for the colors of many fruits and flowers and are proven to be beneficial for human health. They are antioxidants, and they reduce damage caused by free radicals, in addition to having anti-cancer, anti-inflammatory, and anti-viral properties. Moreover, they increase the nutritional value and shelf life of products. Alkaloids, on the other hand, are used in medicine for their sedative, analgesic (pain-relieving), and stimulant effects. However, other alkaloids are toxic and even deadly when taken in high doses. UV light alerts the plant defense system, increases its stress, and reduces growth and yield [64]. This is why only low intensities of UV light in the range of 1–10 μmol/m${}^{2}$s are applied to plants in small durations. The exact values are determined by monitoring plants’ stress when exposing them to different UV light intensities and durations.

## 4. Grow Lights Monitoring System

**Figure 1:**
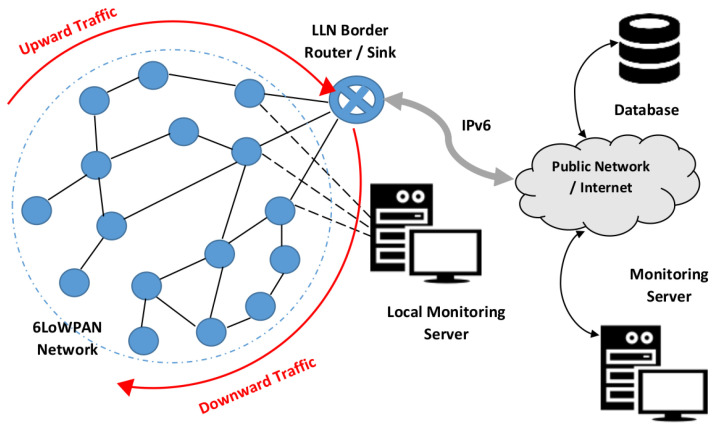
General architecture of a 6LoWPAN network.

Smart farming consists of collecting information about the cultivation process, specifically, collecting data on plants’ environment, including the weather, soil, and light conditions. In addition, data on plant growth and health conditions, and data on the existence of pests, diseases, or herbivores, are gathered. These data are collected by interconnected microcontrollers that are usually equipped with IoT technologies; the data are then analyzed and used to make optimal decisions, automate operations like fertilization and irrigation, improve the products and the productivity, and plan future productions. The general architecture of this IoT-based data collection process is given in Figure 1. The collected data are transferred to the cloud via a gateway where they can be stored, displayed, analyzed, and processed remotely on a distant computing system through the internet, or they can be displayed and processed locally on a server in a control room.

### 4.1. The Network

**Figure 2:**
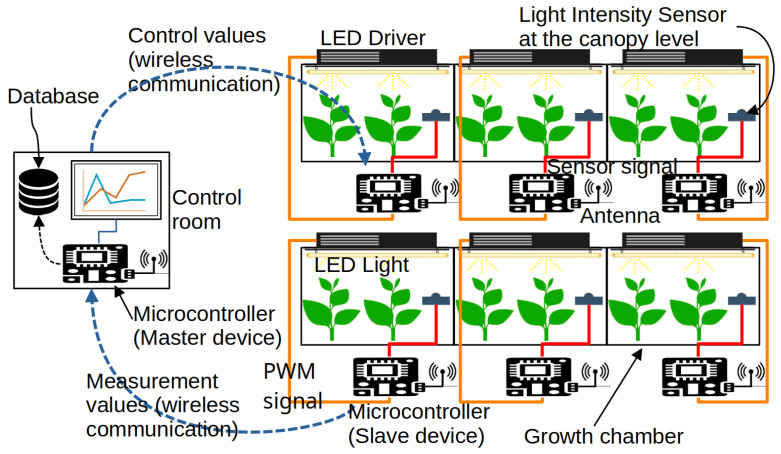
Grow light remote control system for indoor farming. Colored lines represent wired communication, while dashed arrows represent wireless communication.

**Figure 3:**
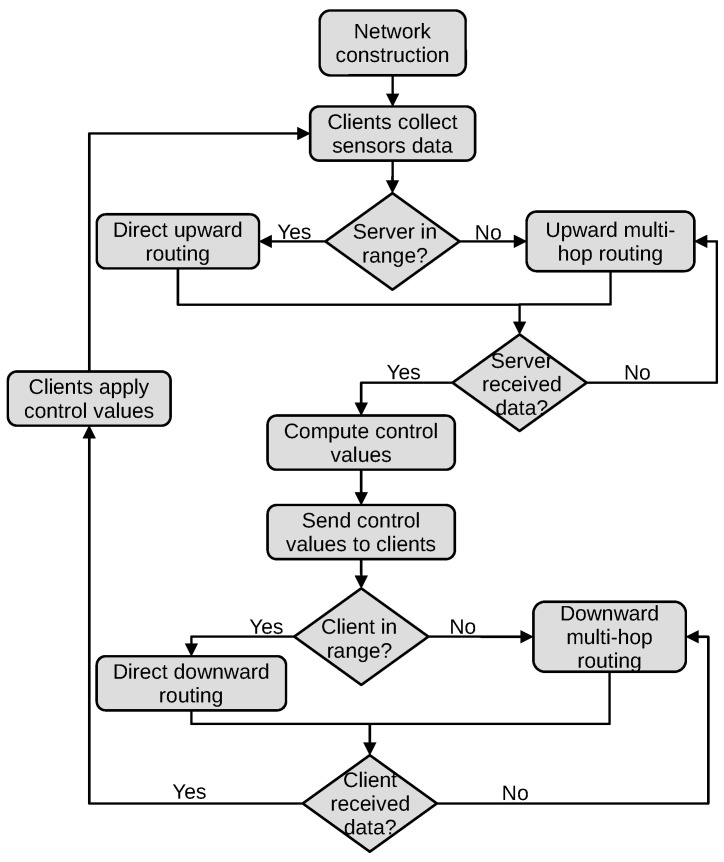
Flowchart of the grow light remote control system for indoor farming.

In order to ensure optimal cultivation conditions, various microcontrollers equipped with sensors, actuators, and wireless communication modules are deployed in the indoor farming facility to monitor and control the system according to the user’s requirements. The indoor farming facility is supposed to be divided into several compartments representing growth chambers and containing different plant species or plants at different growth stages. This means that each growth chamber requires particular environmental conditions and management procedures. The microcontrollers are organized in equidistant positions in a grid topology because the growth chambers are also supposed to be positioned next to each other in a plant factory. On the other hand, a control room is positioned on the side of the growth chambers where another microcontroller, a computer, or a server with the necessary computational power is used for the remote monitoring of the growth conditions. The communication between the microcontrollers is based on the IPv6 protocol, and the range of each microcontroller, including the server, only reaches the neighboring microcontrollers. Multi-hop communication is established to transfer the data between devices out of range. The RPL (routing protocol for low-power and lossy networks) routing protocol works effectively in networks composed of constrained IoT devices used for data collection, generally called WSN (wireless sensor network). Overall, two types of devices exist in the proposed network: a master device and slave devices, also called server and clients. The master device is the root node of the network responsible for processing the data received from other nodes, whereas the slave devices are source nodes that can play two roles: sensing information and forwarding it periodically to the next nodes until reaching the root node, receiving control information from the root and forwarding it to the corresponding sensor node, or applying this information in the growth chamber. Figure 2 illustrates the proposed remote grow light control architecture for indoor farming, and Figure 3 shows the flowchart of the process.

### 4.2. RPL Routing Protocol

RPL is a routing protocol for the networks constrained by their power consumption and packets loss. It constructs the routes towards a single destination node based on a distance vector algorithm minimizing the path length [65]. In addition, it can also use different objective functions (OF) to construct the routes based on other metrics like energy consumption and data packets delivery. RPL supports both upward and downward routing corresponding to sending packets from source nodes to the root node and sending them from the root node to a specific source node, respectively.

Three objective functions are tested in the studied case: OF0 (Objective Function 0), MRHOF-ETX (minimum rank with hysteresis objective function–expected transmission count), and MRHOF-energy (minimum rank with hysteresis objective function–energy). OF0 corresponds to the shortest path, MRHOF-ETX corresponds to the minimum number of transmission attempts per successful transmission (minimum data loss), and MRHOF-energy corresponds to the minimum energy consumption of the nodes. In Section 5, we present the evaluation results of the network performance in terms of data loss, latency, and energy consumption while using these objective functions (OF0, MRHOF-ETX, and MRHOF-energy).

### 4.3. Light Control

LEDs are a sustainable and effective source of artificial light and are continuously gaining ground in indoor farming, to the detriment of the other artificial light sources [66]. Moreover, some LEDs can produce wavelengths that are used in temporary treatments to improve product quality or stimulate the production of important substances with pharmacological properties in medicinal plants. The recent industrial LEDs used in lighting applications can provide higher illuminance than what plants require. In this case, the illuminance of LED lights used in indoor farming has to be dimmed and the light treatments’ duration also has to be controlled to precisely match plants’ requirements. The proposed method to control the grow lights is given in the next sub-sections.

#### 4.3.1. LED Dimming

LED illuminance is controlled by changing the forward current flowing through it. The relationship between the current of an LED and its brightness is approximately linear, contrary to its relation with the voltage. Therefore, an LED driver is used to change the forward current based on either an amplitude modulation (AM) method or a pulse width modulation (PWM) method [67]. The AM method directly changes the amplitude of the forward current, which is easier to control, but it does not ensure efficient dimming, and it changes the chromaticity of the emitted light, which makes it unsuitable for providing the plant light requirements discussed earlier. PWM, on the other hand, changes the duration of the forward current flow through the LED; it does not affect the LED chromaticity since the current amplitude is maintained constant and is therefore suitable for providing the plant light requirements in indoor farming environments. PWM is performed by generating a square-form signal that rapidly switches between the on and off states at a specific frequency. The power delivered to the LED is controlled by changing the on-time of the signal, also called the duty-cycle, which is given by the ratio of on-time in one period of the square signal. The majority of microcontrollers have built-in PWM outputs that can be used to control the brightness of the LEDs only by changing the duty-cycle.

#### 4.3.2. Distributed LED Brightness Control

In the proposed scenario, the LEDs in each chamber of the plant factory or vertical farm are connected to a microcontroller that is responsible for controlling the growth conditions and collecting sensor data in that chamber. Each microcontroller is a node in the network presented earlier, where its role is sending sensor data to the server, receiving back control values from it, and using them to directly adjust the actuators. The microcontroller reads the light intensity value measured by the sensor and sends it to the server for processing. Then, it applies the control value which is the duty-cycle computed by the server and sent back to it in the downward traffic. In the server, a learning algorithm based on the Newton optimization method is implemented to compute the duty-cycles needed to obtain the desired light intensities from the sensor measurements. The cost function minimized in this learning algorithm is given by:

(1) $$J\left(\theta \right)=\frac{1}{2}\sum_{i=1}^{N}{\left(L_{t,i}-{\hat{L}}_{t,i}\right)}^{2}$$

where $L_{t,i}=\theta L_{max,i}$ is the current illuminance at time *t* in growth chamber *i*, θ is the duty cycle, $L_{max,i}$ is the maximum illuminance of the LEDs measured by the sensor, and ${\hat{L}}_{t,i}$ is the desired illuminance in the growth chamber *i* at time *t* fixed by the user. Notice that all these variables ($L_{t,i}$, ${\hat{L}}_{t,i}$, $L_{max,i}$, and θ) are (3 × 1) vectors with three components referring to PAR, far-red, and UV lights, respectively. The Newton method used to update the values of θ and iteratively solve this minimization problem is given by:

(2) $$\theta_{k+1}:=\theta_{k}-\alpha \frac{J^{\prime }\left(\theta_{k}\right)}{J^{\prime \prime }\left(\theta_{k}\right)},\;k=0,1,2,\ldots$$

where α is a learning rate; *k* is the iteration number; and $J^{\prime }\left(\theta \right)=L_{max,i}\left(\theta L_{max,i}-{\hat{L}}_{t,i}\right)$ and $J^{\prime \prime }\left(\theta \right)=L_{max,i}^{2}$ are the first and the second derivatives of $J\left(\theta \right)$ with respect to θ, respectively. A sensor calibration step is implemented in the server, before the learning algorithm, in which the control values of $\theta_{i}=0$ and $\theta_{i}=1$ are sent to each node to read the corresponding values of illuminance $L_{t,i}=0$ and $L_{t,i}=L_{max,i}$, respectively. The implementation of the sensor calibration and the learning method is given in Algorithm 1.

Sensor measurements are noisy, and there is jitter in the packet sending rate. The learning algorithm is not affected by this; it is proven to converge independently of the initial conditions, and sensor noises are averaged out. In addition, updating the value of θ in Equation (2) is performed whenever a new measurement value is received from the client. Thus, jitter does not affect the control method since this is already designed to treat each client separately and wait for packet transmission time that changes depending on the distance from different clients to the server, the route of the data packet, and jitter.
**Algorithm 1** Distributed LED brightness control algorithm1:Initialize $\theta_{i}=0$ for i=1,…,N, α=1, and desired error tolerance ϵ2:*N*: number of clients3:Initialize ${\hat{L}}_{t,i}$ according to user requirements, for i=1,…,N4:#sensor calibration:5:**For** i=1,…,N, **send**$\theta_{i}=0$ to client *i*6:**For** i=1,…,N, **receive** $L_{t,i}=0$ from client *i*7:**For** i=1,…,N, **send** $\theta_{i}=1$ to client *i*8:**For** i=1,…,N, **receive** $L_{t,i}=L_{max,i}$ from client *i*9:#LED brightness control:10:**If** a value $L_{t,i}$ is **received** from a client *i*:11:    **While** *True*:12:        $\theta_{i}:=\theta_{i}-\alpha \frac{J^{\prime }\left(\theta_{i}\right)}{J^{\prime \prime }\left(\theta_{i}\right)}$13:        **If** $J^{\prime }\left(\theta_{i}\right)<\epsilon$:14:            **Break**15:    **Send** $\theta_{i}$ to client *i*16:**Else**17:    **Wait** for $L_{t,i}$ from a client *i*

## 5. Results and Discussion

This section presents the results obtained after implementing the proposed distributed grow light control method using IoT technologies and discusses the performance of the system. Performance evaluation metrics include total energy consumption of the system, data loss ratio, latency, and how they impact the control method used to monitor the lighting conditions in the growth chambers. This allows one to collect information for improving the energy efficiency of plant factories and develop a robust and precise control and monitoring system for indoor agriculture.

### 5.1. Simulation Setup

Simulations were carried out on the network emulator Contiki-NG (next generation), which is an open-source, IoT simulation platform for severely constrained wireless devices. Contiki-NG facilitates the implementation, prototyping, and the evaluation of IoT research works. It reduces the development time of IoT products and allows one to teach IoT systems based on its platform [68]. The main reasons for selecting Contiki-NG are the variety of its new features like the data structure manipulation libraries and the network evaluation tools. The simulations were performed under the conditions listed in Table 1.

**Figure 4:**
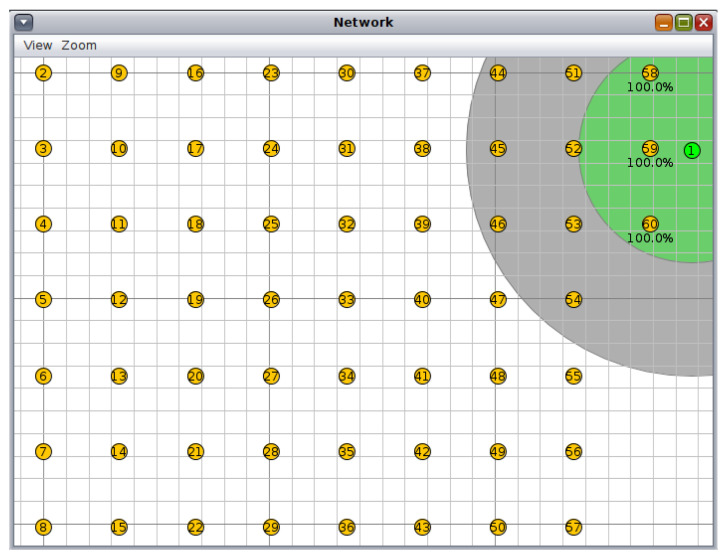
The network simulation in the 60 nodes case; the first node is the server, and the other nodes are clients.

The proposed 6loWPAN network consists of 10 to 60 nodes in which client nodes are distributed in a grid topology with equal distances between the nodes in each direction, whereas the root of the network is located on the side of the grid. Figure 4 shows the nodes’ positions as they are supposed to be in the indoor farming facility. The server node is the first node in the network (green color), and the remaining nodes (yellow color) are the clients. Each client node is responsible for controlling the growth conditions—more specifically lighting—in a growth chamber, so the number of nodes represents the number of the growth chambers, and the size of the indoor farming facility can be estimated based on it.

The nodes used in the simulation are the Zolertia Z1 motes (https://github.com/contiki-os/contiki/wiki/Zolertia-z1-motes (accessed on 20 March 2023)) generally used in WSN. They are equipped with the second generation of Texas Instruments’ MSP430F2617 low-power and efficient microcontroller (https://www.ti.com/product/MSP430F2617 (accessed on 20 March 2023)) containing a 16-bit RISC CPU functioning at 16 MHz clock speed, a built-in clock, an 8 KB RAM, and a 92 KB Flash memory. They are also equipped with the CC2420 transceiver, which operates at 2.4 GHz and has a data rate of 250 Kbps.

### 5.2. Distributed Remote Control Results

**Figure 5:**
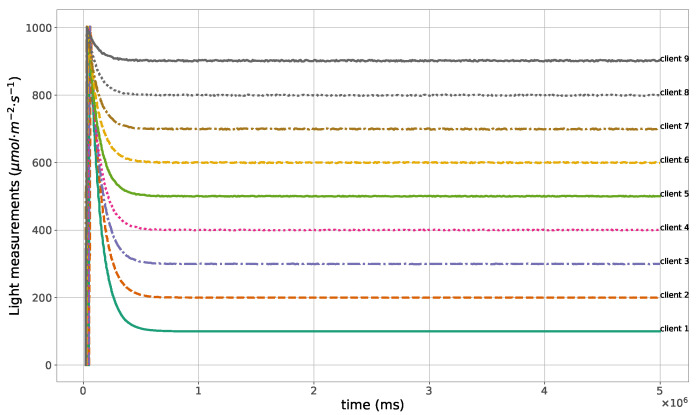
PAR light measurements sent from the clients to the server.

**Figure 6:**
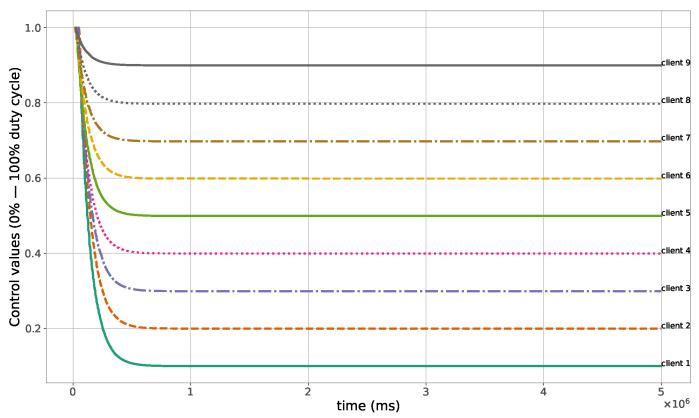
PAR light control values sent from the server to the clients.

**Figure 7:**
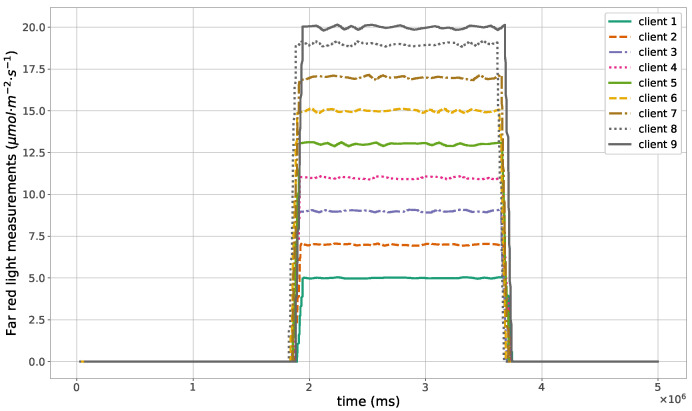
Far-red light measurements sent from the clients to the server.

**Figure 8:**
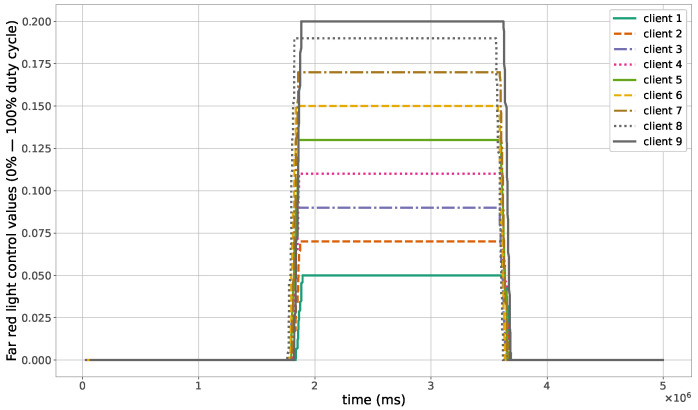
Far-red light control values sent from the server to the clients.

**Figure 9:**
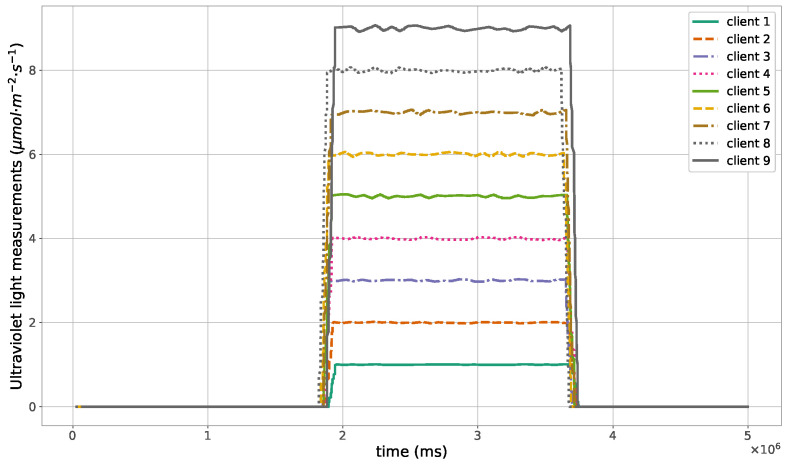
Ultraviolet light measurements sent from the clients to the server.

**Figure 10:**
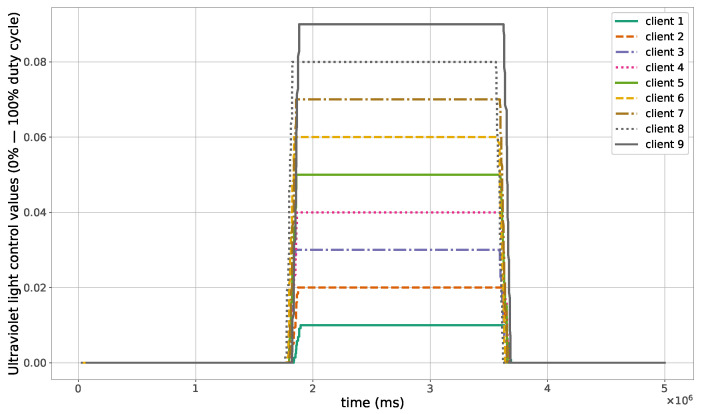
Ultraviolet light control values sent from the server to the clients.

**Figure 11:**
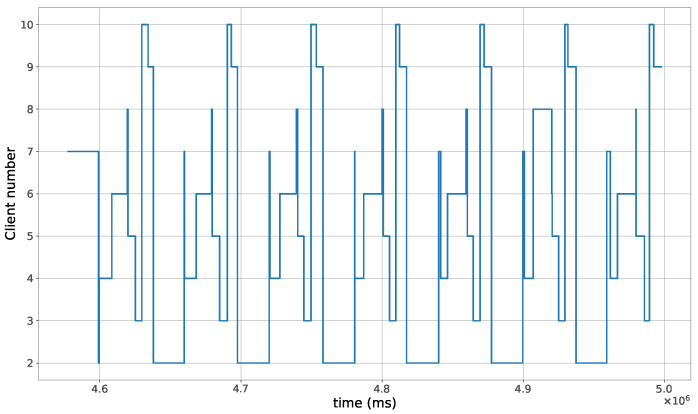
The communication sequence to the server during the light control process.

The proposed method for the remote distributed control of grow lights was programmed in C language and implemented in the root node. Three different types of LED lights were considered in each growth chamber: PAR lights with intensity ranging from 100 to 900 μmol · m${}^{-2}$·$s^{-1}$, far-red lights with intensity ranging from 5 to 20 μmol · m${}^{-2}$·$s^{-1}$, and UV lights with intensity ranging from 1 to 9 μmol · m${}^{-2}$·$s^{-1}$. This range of different values was used to test the control method; they are not exact values, but they are within the range of values needed by some plants grown in indoor environments. The maximum illuminance produced by these LED lights is supposed to be equal to 1000 μmol · m${}^{-2}$·$s^{-1}$. The network was then simulated under the previously mentioned conditions, and the obtained results in the case of 10 nodes are given in Figure 5, Figure 6, Figure 7, Figure 8, Figure 9, Figure 10 and Figure 11. Figure 5 is produced by the server, and it shows the PAR light intensities measured by the sensors connected to the clients in the 9 growth chambers of the indoor farming facility. The measurements are noisy, and it can be noticed that they rapidly converge to the desired values. The control values sent from the server to the clients to obtain the previous measurements are given by Figure 6; notice that these values are not affected by sensor noises. Similarly, Figure 7 and Figure 9 are produced by the server, and they show the far-red and UV light intensities measured by the clients in the 9 growth chambers, respectively. It can be noticed that the control method also works for these types of temporary and low intensity light treatments. The light intensities converge to the desired values, and despite the fact that the treatments do not start at the same time in all the growth chambers, they all have the same duration as fixed from the server, which is the most important for plants. These different starting times of the light treatments are negligible (in milliseconds) and depend on the routes chosen by the routing protocol based on the selected OF, the topology of the nodes, data scheduling, and the performance of the network. The control values of far-red and UV lights sent from the server to the clients that led to one obtaining these measurements are given in Figure 8 and Figure 10, respectively. It is worth mentioning that, due to data loss that can occur during communication, some values are missing and are replaced by interpolation so that the previous figures can be obtained from the recipient side. Figure 11 shows the communication sequence between the clients and the server during this light control process; this can change depending on the topology of the nodes, the scheduling algorithm, and the route chosen by the routing protocol depending on the used OF. This figure can give us an intuition about why the far-red and UV light treatments do not start at the same time in all the growth chambers.

### 5.3. Network Performance Evaluation

**Figure 12:**
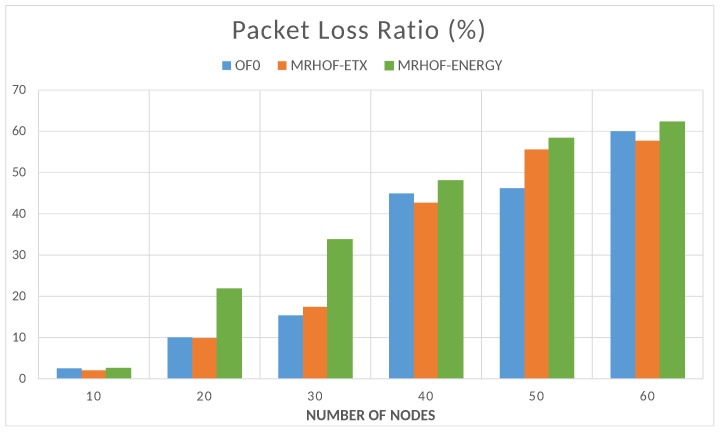
Packet loss ratio depending on the size of the network.

After showing that the control method works, it is necessary to see if the data transmission in the constructed network is good enough to ensure that the measured sensor values and the computed control values reach their destination and prove the efficiency of the overall system. The network was evaluated in terms of data transmission quality, including the amount of packet loss and the delay in packet delivery. It was also evaluated in terms of the energy consumed by the nodes for computation, data sending, and listening (waiting for data). To do so, the system was simulated for 10,000 s under the conditions listed in Table 1, and the evaluation metrics were computed for a network size varying from 10 to 60 nodes. The packet loss ratio (PLR) is computed by Equation (3):

(3) $$PLR=\left(1-\frac{\sum Received\;packets}{\sum Sent\;packets}\right)100$$

the obtained results are shown in Figure 12, where it can be seen that the packet loss ratio increases with the network size. This graph shows that there is no big difference between the three RPL OFs, but the MRHOF-energy OF shows the highest packet loss ratio because it basically chooses the forwarding nodes based on their energy consumption and not based on the success of packet transmission like the MRHOF-ETX.

**Figure 13:**
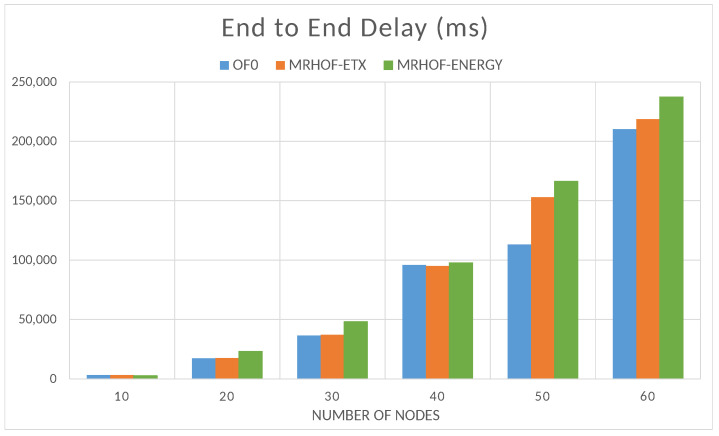
Accumulated end-to-end delay depending on the size of the network.

The end-to-end delay or the latency is computed by Equation (4):

(4) $$Delay=\sum_{i=1}^{n}\left(RT_{i}-ST_{i}\right)$$

where $RT_{i}$ is the packet receiving time of the node *i* and $ST_{i}$ is its packet sending time. Similarly, the time of packet delivery also increases with the network size; this is because the packets pass through a higher number of forwarding nodes before reaching their destination as shown in Figure 13. The objective function OF0 performs better in this case since it is designed to choose the shortest path to the destination, which reduces the number of forwarding nodes for the packets.

**Figure 14:**
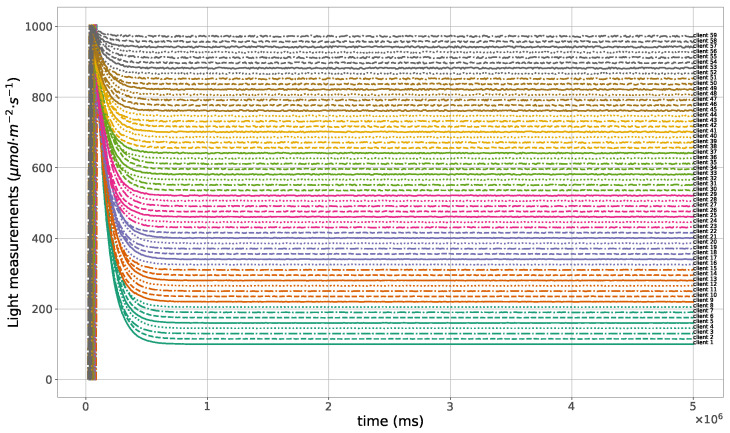
PAR light measurements sent from the clients to the server, 60 nodes case.

**Figure 15:**
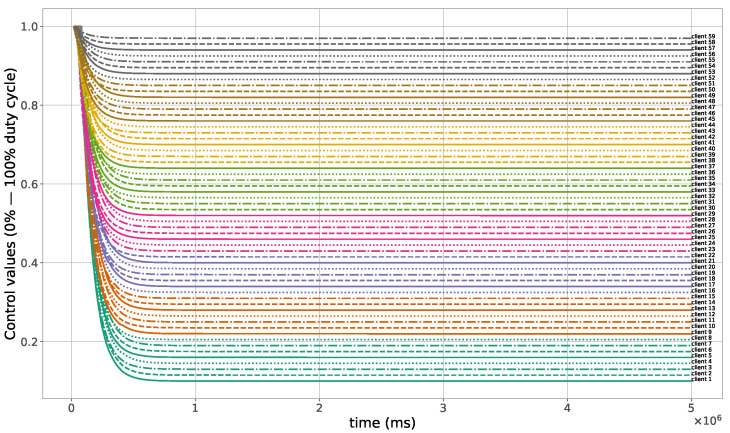
PAR light control values sent from the server to the clients, 60 nodes case.

A high packet loss ratio and delay can occur in the case of large size networks; see, for instance, the 60 nodes case in Figure 12 and Figure 13. To see if this affects the control method and changes the light conditions in the growth chambers, another simulation with 60 nodes was performed. The obtained light measurements that were sent from the clients and received by the server are shown in Figure 14, while the control values sent from the server to the clients are plotted in Figure 15. Notice that the control process was not affected in this case, and all the measured light intensities converge to the desired values.

**Figure 16:**
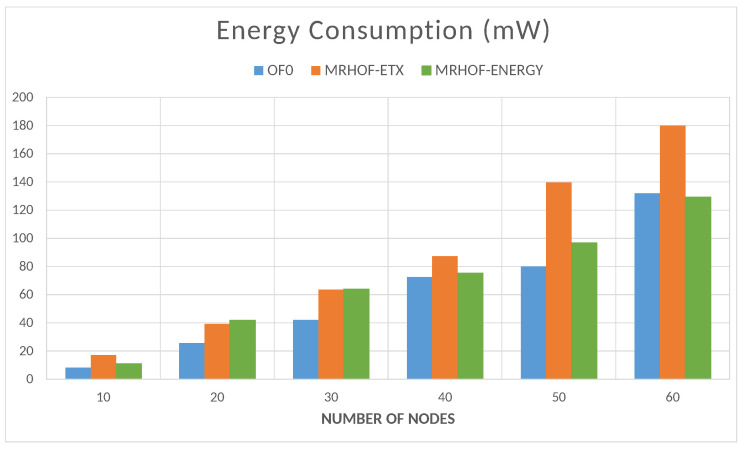
Total energy consumption of all the nodes depending on the size of the network.

**Figure 17:**
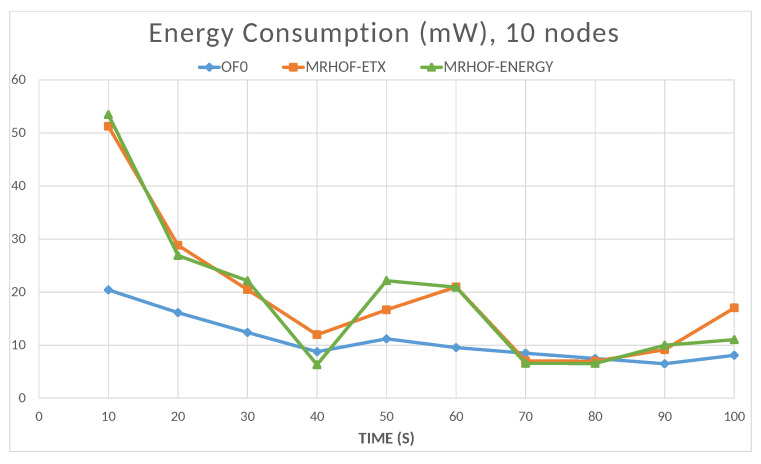
Total energy consumption of all the nodes depending on the simulation time (10 nodes case).

**Figure 18:**
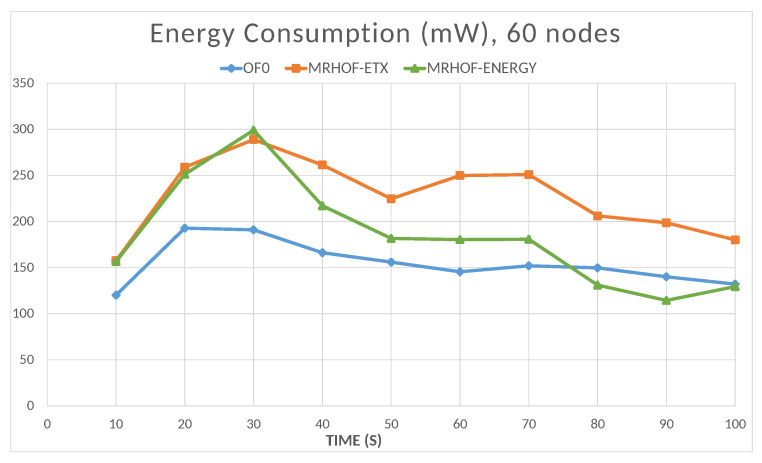
Total energy consumption of all the nodes depending on the simulation time (60 nodes case).

The energy is computed by the Energest tool (Powertrace in Contiki); the resulting energy consumption value is the sum of the amounts consumed by the microcontroller in the CPU processing mode, CPU low-power mode, data transmission mode, and data listening mode. The total energy consumption of the network naturally increases with the number of nodes, as shown in Figure 16. In this case, the objective functions OF0 and MRHOF-energy perform better than MRHOF-ETX. The reasons for this are that the MRHOF-ETX calculation method is computationally more expensive than the other OFs and may consume more energy; also, the routes chosen by the MRHOF-ETX are not necessarily the shortest ones but the ones that ensure the packet delivery. Figure 17 and Figure 18 show the evolution of the energy consumption over time for the 10 and 60 nodes cases, respectively. It can be seen that the energy consumption stabilizes after a certain time when the network configuration is finished, the routes are established, and the computation time spent on the routing process decreases. For small networks (Figure 17), all the OFs give the same performance after network stabilization, in terms of energy consumption, while the difference between them appears in large networks (Figure 18), where the previous observation on the high energy consumption of MRHOF-ETX is confirmed.

## 6. Conclusions

The benefits and the drawbacks of smart indoor agriculture were discussed in this article, with more emphasis given to its energy consumption as its biggest disadvantage. The research was focused on tools and methods to reduce its energy consumption. Preliminary studies discussed the choice of hardware like energy-efficient LEDs instead of the other types of grow lights, PWM LED drivers that allow one to reduce the intensity of the emitted light without affecting its quality, and low-power microcontrollers to collect sensor data and control the LED drivers according to the user’s requirements. The main contribution is focused on the software part of the problem, where a low-computational-cost control algorithm is proposed based on the Newton method, which iteratively updates the control values only when the light intensity is different from the desired one. Furthermore, energy-efficient methods for data transmission are used, allowing one to easily monitor the growth conditions by using wireless communication technologies and implementing communication protocols like RPL that are designed for constrained networks. The results showed that the control method performs well in small- and large-scale plant factories, and the network evaluation results showed that the data transmission quality decreases when the network size is large and when trying to reduce the energy consumption of the network by considering the MRHOF-energy objective function. Yet, it has been shown that, despite this decrease in the data transmission quality, which occurs only in extreme communication conditions when the devices are out of the range, the control method was still able to ensure the proper management of the light conditions, even in large-scale plant factories. It can be concluded that improving the energy efficiency of the production process while ensuring optimal growth conditions for plants is still possible and appears to be the best solution for future food security challenges. Different methods for the wireless management of all the growth factors, including light, temperature, humidity, ventilation, and substrate quality to maximize the plants yields in plant factories, could be the subject of future research.

## Figures and Tables

**Table 1:** Network simulation conditions.

Parameter	Value
Operating system/simulator	Contiki-NG
MAC layer	IEEE 802.14.5
Network type/addressing scheme	6LoWPAN/IPv6
Transport	UDP
Radio medium model	Unit disk graph medium (UDGM): distance loss
Area	100×100,…,300×300 m^2^
Number of nodes	10,…,60
Simulation time	5,000,000 ms
Objectives functions	OF0, MRHOF (ETX, Energy)
Transmit/receive ratio	TX = 100%, RX = 100%
Transmission range	50 m
Interferance range	100 m
Topology	Grid, multipoint-to-point; point-to-point
Nodes type	Zolertia Z1
Packet sending rate (from clients)	1 packet/s

## Data Availability

No new data were created or analyzed in this study. Data sharing is not applicable to this article.

## Abbreviations

The following abbreviations are used in this manuscript:

IoT	Internet of things
AI	Artificial intelligence
LEDs	Lighting-emitting diodes
PAR	Photosynthetically active radiation
UV	Ultraviolet
IPv6	Internet protocol version 6
6LoWPAN	IPv6 over low-power wireless personal area networks
RPL	Routing protocol for low-power and lossy networks
PWM	Pulse width modulation
AM	Amplitude modulation
PI	Proportional integral
PFD	Photon flux density
WSN	Wireless sensor network
OF	Objective function
OF0	Objective function 0
MRHOF-ETX	Minimum rank with hysteresis objective function-expected transmission count
MRHOF-energy	Minimum rank with hysteresis objective function-energy
PLR	Packets loss ratio
